# Evaluation of Phosphate Fertilizers for the Immobilization of Cd in Contaminated Soils

**DOI:** 10.1371/journal.pone.0124022

**Published:** 2015-04-27

**Authors:** Yin Yan, Yi Qun Zhou, Cheng Hua Liang

**Affiliations:** Department of Land and Environment, Shenyang Agricultural University, Shenyang, Liaoning, China; University of Vigo, SPAIN

## Abstract

A laboratory investigation was conducted to evaluate the efficiency of four phosphate fertilizers, including diammonium phosphate (DAP), potassium phosphate monobasic (MPP), calcium superphosphateon (SSP), and calcium phosphate tribasic (TCP), in terms of the toxicity and bioavailability of Cd in contaminated soils. The efficiency of immobilization was evaluated on the basis of two criteria: (a) the reduction of extractable Cd concentration below the TCLP regulatory level and (b) the Cd changes associated with specific operational soil fractions on the basis of sequential extraction data. Results showed that after 50 d immobilization, the extractable concentrations of Cd in DAP, MPP, SSP, and TCP treated soils decreased from 42.64 mg/kg (in the control) to 23.86, 21.86, 33.89, and 35.59 mg/kg, respectively, with immobilization efficiency in the order of MPP > DAP > SSP > TCP. Results from the assessment of Cd speciation via the sequential extraction procedure revealed that the soluble exchangeable fraction of Cd in soils treated with phosphate fertilizers, especially TCP, was considerably reduced. In addition, the reduction was correspondingly related to the increase in the more stable forms of Cd, that is, the metal bound to manganese oxides and the metal bound to crystalline iron oxides. Treatment efficiency increased as the phosphate dose (according to the molar ratio of PO_4_/Cd) increased. Immobilization was the most effective under the molar ratio of PO_4_/Cd at 4:1.

## Introduction

Heavy metal contamination in soils is undoubtedly one of the major issues in modern soil science. Such heavy metal contamination may adversely affect water resources, soil ecology, and agricultural product quality while causing serious health problems for humans and animals. Mining, processing, and smelting activities, industrial wastes, as well as sewage irrigation are all major Cd sources [1]. Therefore, increasing awareness of the public health hazard presented by Cd has necessitated the restoration of Cd-contaminated soils.

The demand for Cd immobilization has increased with its increasing content in soil environment over the last decades [2]. Among the varieties of promising remediation technologies [3–8], in situ chemical immobilization is an effective remediation technology that may reduce the extractable potential of heavy metals. Furthermore, phosphate fertilizers are considered as cost-effective materials for immobilization. The addition of phosphate fertilizers can cause a reaction between metal ions in soils and phosphate [9–11]. Fertilizers could alter soil characteristics, such as pH, available phosphate, and surface charge, or could directly react with metal ions in soils. As a result, phosphate treatment can induce a shift in the mobile forms of heavy metals toward more stable forms. The aim of immobilization is the reduction in the bioavailable fraction of metals, either through increased metal sorption or precipitation or through the formation of discrete minerals [12–14].

This immobilization mechanism has recently become widely used in the case of Pb-contaminated soils, and phosphates have served an important function in the immobilization of Pb-contaminated soils. Although phosphate amendments have mainly been applied to remediate Pb-contaminated soil, these alterations may also be applicable to other metals, such as Cd [15], which frequently occur simultaneously with Pb in soils. Nevertheless, the effectiveness of the use of phosphate to immobilize other metals, such as Cd, and its mechanisms remain poorly understood.

Research on Cd immobilization with various phosphate fertilizers is still needed, this work thus aims to evaluate the efficiency of four different kinds of phosphate fertilizers, namely, diammonium phosphate (DAP), potassium phosphate monobasic (MPP), calcium superphosphateon (SSP), and calcium phosphate tribasic (TCP), for the immobilization of Cd in contaminated soils. Factors affecting treatment, such as phosphate dose and immobilization time, were also analyzed.

## Materials and Methods

### 2.1. Soil preparation and characterization

The soil used was collected from meadow soil, Yuhong District, Shenyang City, Liaoning Province, China (latitude 41°39′N, longitude 123°05′E). The soil was classified as loam [16]. The amount of total Cd was 0.07 mg/kg, which exceeded the allowed figure. The experiment was initiated in 2013. Before this period, the field was an open vegetable plot. No specific permissions were required for this location. In October 2012, the soil sample was excavated from the surface layer (0–0.2 m), transferred to plastic bags, and transported to the laboratory. The samples were mixed prior to analysis. The air-dried samples were passed through a 2 mm sieve, and the visible crop residue and roots were removed. The most important characteristics are presented in Table 1.

**Table 1:** Characterization of the soil in the experiments.

pH	OM (g/kg)	Sand fraction (%)	Silt fraction (%)	Clay fraction (%)	Available phosphate(mg/kg)	Total Cd (mg/kg)
5.39	19.8	49.2	25.1	25.7	18.04	0.07

The soil was prepared by adding cadmium nitrate solution (AR grade) to air-dried soil to yield a concentration of approximately 100 mg/kg. The soil was mixed to achieve homogeneity and then left to stand at 25°C for a week. The soil was then air-dried and stored in plastic containers for physical and chemical characterization, as well as subsequent immobilization tests.

### 2.2. Chemical immobilization treatments

Four chemical treatment materials, namely, DAP, MPP, SSP, and TCP, were evaluated in terms of TCLP extractable Cd extraction. The phosphate dose was calculated on the basis of the molar ratio of PO_4_^3-^ in fertilizer to the total concentration of Cd in soil, i.e., 100 mg Cd per kg of soil. The addition of phosphates at a molar ratio 2 mole/mole corresponds to 4.7 g of DAP per kg of soil for this particular soil sample. Each immobilization test included 50 g soil, a predetermined weight ratio of phosphate fertilizer to dry soil weight, and amounts of water sufficient to reach soil saturation level. All materials were thoroughly mixed in a plastic container. Five molar ratios of PO_4_/Cd were tested: 0 (no addition), 2:3, 3:2, 2:1, and 4:1. Soil samples were collected five times over a 50 d testing period. To promote the chemical reactions between Cd ions retained in soil and the phosphate additive, the containers were systematically hydrated and kept saturated for approximately 50 d.

### 2.3. Chemical tests

Following soil treatment with phosphate fertilizers, representative samples from each container were subjected to several leaching tests to evaluate the efficiency of phosphates for Cd immobilization, as well as Cd availability.

#### 2.3.1. TCLP leaching test

The TCLP test used in this study was modified from the original methods described by the USEPA (1986). The modified TCLP method is based on soil pH and buffer capacity to develop two kinds extracting solution. No. 1 Extracting solution (used when pH < 5): A 5.7 ml aliquot of glacial acetic acid and 64.3 ml aliquot of sodium hydroxide were diluted to 1.0 L with deionized water. The pH of the extracting solution was 4.93±0.05. No. 2 Extracting solution (used when pH>5): A 5.7 ml aliquot of glacial acetic acid was diluted to 1.0 L with deionized water. The pH of the extracting solution was 2.88±0.05. A 20 ml extracting solution was added to a 50 ml polycarbonate centrifuge tube containing 1.000 g of test soil. The tube was sealed with a cap and placed on an end-over-end shaker at 30±2 r/min for 18 h at 25°C. After extraction, separation was performed via centrifugation (J2-21, Beckman, USA) at 4,000 r/min for 30 min. The buffer solution pH was adjusted using 1 mol·L^-1^ HNO_3_ and 1 mol·L^-1^ NaOH.

#### 2.3.2. Sequential extraction analysis

The sequential extraction developed by Tessier et al. [17] was used to estimate the availability and chemical speciation of Cd in treated and untreated soils. This procedure was developed for tropical soils and extracts metal associated with five fractions: (1) soluble—exchangeable, (2) bound to carbonates or weakly specifically adsorbed, (3) bound to Fe-Mn oxides, (4) bound to organic matter, and (5) residual. *S*equential extractions were performed in triplicate using 1 g of air-dried soils. Soil samples were then placed in 50 ml polycarbonate centrifuge tubes and mixed with various reagents in a step-wise manner. The suspensions were equilibrated as described in Table 2.

**Table 2:** Sequential extractions steps for Cd in contaminated soils.

Step	Fraction	Extraction procedure
1	Soluble-exchangeable (SE)	8 ml 1M MgCl_2_ (pH7,2 h, room temperature)
2	Bound to carbonates (WSA)	8 ml 1 M NaAc (pH5,5 h, room temperature)
3	Bound to Fe-Mn oxides (OX)	20 ml 0.04 M NH_2_OH·HCl (pH 2, 6 h, 96°C)
4	Bound to organic matter(OM)	3 ml 0.02 M HNO_3_+5 ml 30% H_2_O_2_ (pH 2, 2 h, 85°), 3 ml 30% H_2_O_2_ (3 h, 85°C), 5 ml 3.2 M CH_4_COONH_4_ (30 min, room temperature)
5	Residual (RES)	15 ml HF–3 ml HClO_4_ digestion

#### 2.3.3. Analytical methods

All extractions of treated samples were conducted in triplicate in acid-washed (5% HNO_3_) polycarbonate centrifuge tubes and filtered through a 0.2 μm membrane (Schleicher & Schuell, Germany). All chemicals used in this study were of analytical grade or better. Double deionized water was used. Soil pH was measured in 1:2.5 soil/water (v/w) suspensions with PHS-3C. Available phosphate in soil was determined by the colorimetric method using Olsen solution [18]. Total cadmium concentrations in soil were determined through a total microwave digestion of 0.5 g of soil with concentrated acids (10 ml of HNO_3_ and 5 ml of HClO_4_). The resulting extracts, as well as those from the sequential extraction procedures, and soil leachates from TCLP were filtered through 0.45 μm membrane filters, acidified with HNO_3_, and stored at 2°C to 5°C until analysis. Cd concentrations were analyzed by flame atomic absorption spectrophotometer (Flame AAS, AA320N).

## Results and Discussion

### 3.1 Characterization of phosphate fertilizers in the study

The chemical properties of phosphate fertilizers used in the experiment are presented in Table 3. TCP is insoluble in water as compared with three other phosphate fertilizers

**Table 3:** Properties of phosphate fertilizers in the study.

Phosphate reagent	Chemical composition	Abbreviation	Solubility
Diammonium Phosphate	(NH_4_)_2_HPO_4_	DAP	Water-soluble
Potassium Phosphate Monobasic	KH_2_PO_4_	MPP	Water-soluble
Calcium Superphosphateon	Ca(H_2_PO_4_)·CaSO_4_·H_2_O	SSP	Water-soluble
Calcium Phosphate Tribasic	Ca_3_(PO_4_)_2_	TCP	Water-insoluble

### 3.2 Treatment of different phosphate fertilizers

**Fig 1:**
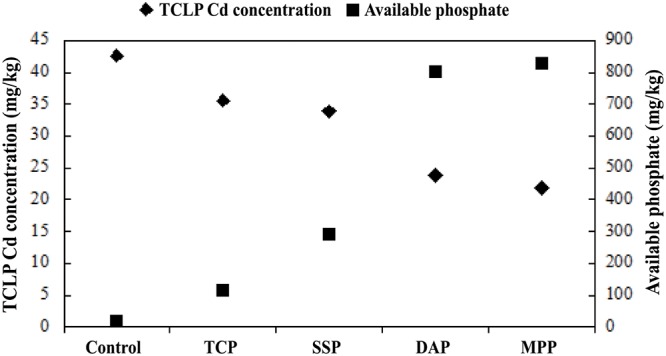
TCLP extractable Cd concentration and soil available phosphate in the control and phosphate treated soils after 50 d immobilization.

The influence of phosphate treatment on TCLP extractable Cd and soil-available phosphate is shown in Fig 1. TCLP extractable Cd concentration can be significantly decreased after application of TCP, SSP, DAP, and MPP treatment to the contaminated soils with reductions of 16.52%, 20.51%, 44.05%, and 48.73%, respectively, as compared with the control sample. Variation in efficiency immobilization is attributed to the available phosphate released by each phosphate fertilizer. As shown in Fig 1, treating the soil through the application of phosphate causes a significant increase in available phosphate. The available phosphate concentration increased from 18.04 (controlled) to 113.55, 291.21, 803.02, and 827.61 mg/kg after 50 d immobilization with TCP, SSP, DAP, and MPP, respectively. Among the used phosphate fertilizers, MPP and DAP are pure phosphorus reagents, whereas SSP and TCP are commercial reagents, thus making SSP and TCP less reactive than MPP and DAP in supplying available phosphate to stabilize Cd. Moreover, an influence from calcium ion on the stability of Cd was seen. The important role of Ca^2+^ in Cd immobilization has been proved by many authors [19, 20].

**Fig 2:**
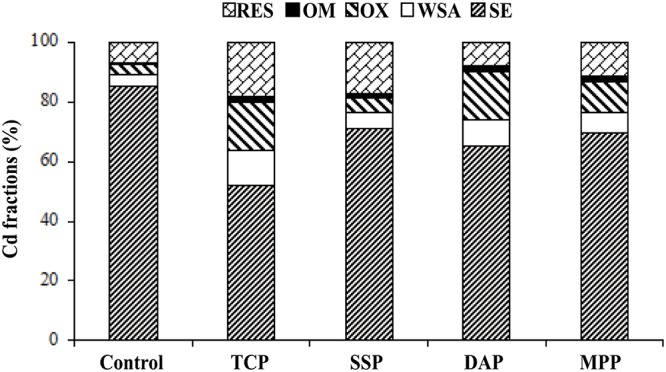
Effect of phosphate addition on fractions of Cd after 50 d immobilization.

To investigate the efficiency of applying phosphate fertilizers and to show the effect of phosphate addition on fractions of Cd in soils, sequential extraction analysis was performed. All phosphate additions resulted in a significant decrease in the SE fraction of Cd, and applying phosphate fertilizers increased the other four fractions of Cd. However, this effect was not significant (Fig 2). These findings imply that phosphate fertilizers may be a “bridge” between soil and sorbed heavy metals [21]. The SE fraction of Cd concentration decreased from 85.25 mg/kg (in the control) to 51.96, 71.10, 65.00, and 69.60 mg/kg after 50 d immobilization with TCP, SSP, DAP, and MPP, respectively. Compared with the control, the addition of all used phosphate fertilizers can cause the conversion of mobile forms of Cd into more stable forms.

### 3.3 Dosage of different phosphate fertilizer treatments

**Fig 3:**
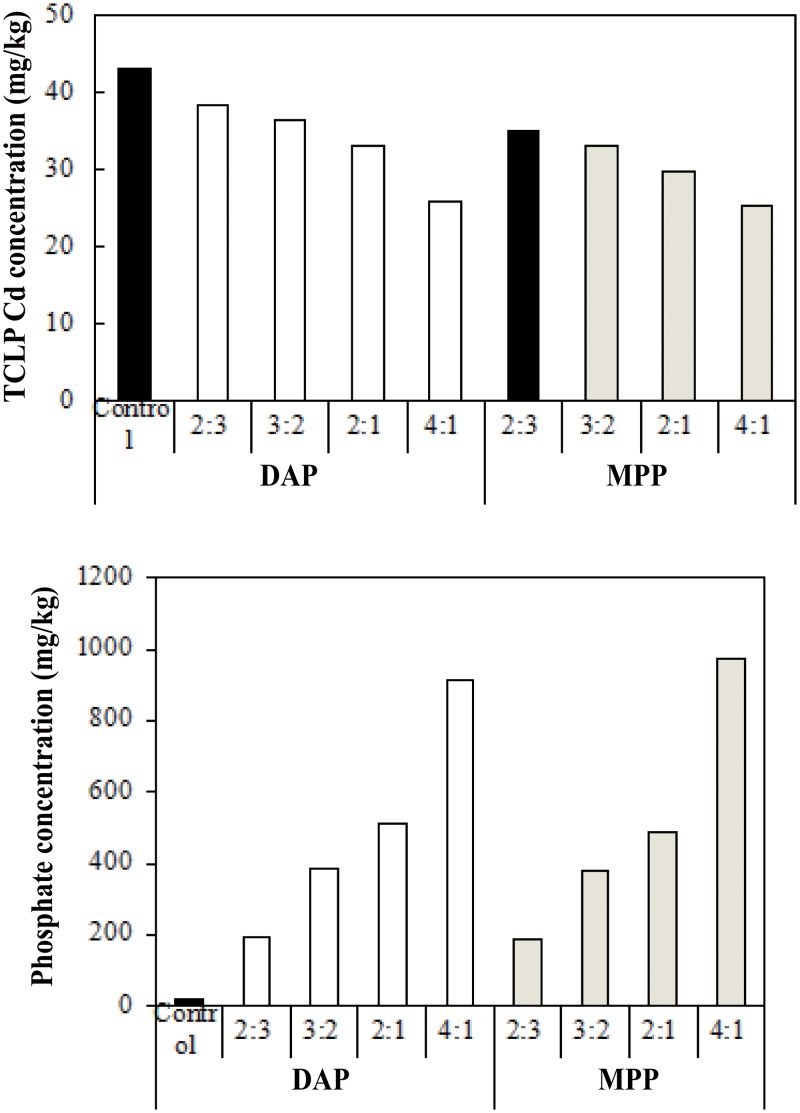
TCLP extractable Cd concentration and soil available phosphate at various phosphate doses after 28 d immobilization.

The effect of phosphate fertilizer dose on Cd immobilization was tested on two phosphate fertilizers, namely, DAP and MPP, which effectively decrease the TCLP extractable Cd concentration at 44.05% and 48.73% reductions, respectively (Fig 1). The variation of Cd leachability as a function of phosphate dose is presented in Fig 3. As a result, the TCLP extractable Cd concentration in soils with DAP and MPP amendment decreased after 28 d immobilization as phosphate dose (based on the molar ratio of PO_4_^3-^ to Cd) increased from 0 (no addition) to 2:3, 3:2, 2:1, and 4:1. The best treatment efficiency was under the PO_4_/Cd molar ratio of 4:1, and the greatest Cd reduction values were 39.70% and 41.41% (Fig 3). A comparison between DAP and MPP at the most effective dose at 4:1 revealed that MPP was more effective than DAP because the TCLP extractable Cd concentration in MPP-treated soils was lower than that in DAP-treated soils. This finding may be attributed to the soil available phosphate concentration released by fertilizers. It was also found that N fertilization increased the bioavailable of Cd and reduced Cd accumulation [22, 23]. A larger amount of MPP than DAP resulted in greater available phosphate of fertilizers to stabilize Cd in soil. The application of phosphate was found to increase the available phosphate efficiently. Furthermore, the change in soil available phosphate was significant when the phosphate dose was increased (Fig 3). The results suggest that TCLP extractable Cd concentration was significantly negatively correlated with soil available phosphate concentration.

**Fig 4:**
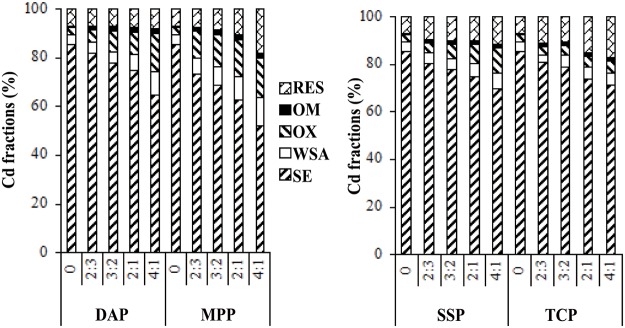
Effect of phosphate addition on fractions of Cd after 28 d immobilization.

The results of sequential extraction of Cd are shown in Fig 4. Both phosphate amendments (DAP and MPP) resulted in a significant shift in the mobile forms of Cd to stable forms. Fig 4 shows the changes in the fractions of five operationally defined Cd species in the soils treated with five different doses of fertilizers. In the control sample, the most abundant fraction of Cd was the soluble—exchangeable (SE) fraction, which was 87.90 mg/kg (87.9% of the total Cd content). This result is in line with that of Sungur et al. [19] who found that the quantity of the mobile fractions of Cd was higher than that of the immobile fractions. Compared with that in the control sample, the Cd concentration of SE was decreased, whereas an increase in Cd associated with the four other fractions was observed in both DAP and MPP treated soils. Furthermore, the shift of Cd speciation was obviously observed once the phosphate dose (based on the molar ratio of PO_4_ to Cd) increased from 0 (no addition) to 2:3, 3:2, 2:1, and 4:1.

### 3.4 Treatment of immobilization time

**Fig 5:**
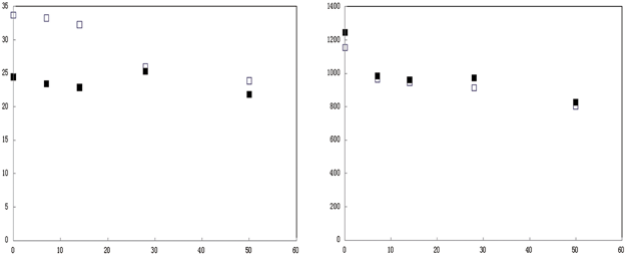
TCLP extractable Cd concentration (left) and soil available phosphate (right) in DAP treated soils and MPP treated soils.

The TCLP extractable Cd concentration in DAP and MPP treated soils at various times is presented in Fig 5. The extractable Cd concentration and soil available phosphate concentration in DAP treated soil decreased with increasing immobilization time from 0 d to 50 d. However, the extractable Cd concentration and soil available phosphate concentration in MPP treated soil initially decreased, but then increased at 28 d, after which it again decreased. The greatest treatment efficiency was achieved at immobilization time of 50 d. Compared with DAP, MPP was more effective as a stabilizing agents.

## Conclusions

This research showed that the extractable Cd concentration decreased when contaminated soil was immobilized with phosphate fertilizers. Extractable Cd concentration was reduced, as evidenced by the TCLP test and sequential extraction analysis. Of all the treatments, MPP was the most effective stabilizing agent with the highest reduction (48.73%) of Cd concentration compared with the control. The addition of MPP can result in the greatest release of soil available phosphate, which mainly influences immobilization efficiency. DAP was highly effective in stabilizing Cd extracted from contaminated soil with a reduction of 44.05%. It was also found that N fertilization increased the bioavailable of Cd and reduced Cd accumulation. By contrast, SSP and TCP exhibited low efficiency. Potential negative effect of Ca^2+^ due to fertilizers on Cd stability was found. Both of MPP and DAP were highly effective because they were pure phosphate fertilizers. Meanwhile, SSP and TCP were commercial fertilizers. Pure fertilizers released more soil available phosphate concentration than commercial fertilizers. The extractable Cd concentration decreased by increasing phosphate addition dose. The greatest immobilization dose was found to be at 4 molar PO_4_ to 1 molar Cd. Although DAP and MPP are highly effective in reducing extractable Cd concentration, the addition of both fertilizers did not result in P elution in this work. Co-additions of limiting materials with phosphate fertilizers may be essential to offset potential soil acidification.

## Supporting Information

S1 File TCLP extractable Cd concentration and soil available phosphate in the control and phosphate treated soils after 50-day immobilization. (XLS)

S2 File Effect of phosphate addition on fractions of Cd after 50-day immobilization. (XLS)

S3 File TCLP extractable Cd concentration and soil available phosphate at various phosphate doses after 28-day immobilization. (XLS)

S4 File Effect of phosphate addition on fractions of Cd after 28-day immobilization. (XLS)

S5 File TCLP extractable Cd concentration and soil available phosphate in DAP treated soils and MPP treated soils. (XLS)
